# Atlanta Society of Medicine

**Published:** 1899-11

**Authors:** 


### *SOCIETY REPORTS.*

### ATLANTA SOCIETY OF MEDICINE.

ATLANTA, GA., September 21, 1899.

*Regular Meeting of the Atlanta Society of Medicine.*

The meeting was called to order by the President, Dr. W. S. Goldsmith.

Dr. Dunbar Roy read a paper on "Subconjunctival Injections in Certain Diseases of the Eye."

### DISCUSSION.

*Dr. Duncan:* I consider the paper of Dr. Roy one of the best ever heard on ophthalmology. I consider it well timed, and it brings out many points that are of great practical utility to the oculist. I am sorry there are no oculists here to discuss it.

Dr. M. B. Hutchins read a paper on "Conditions Successfully Treated by Electrolysis."

### DISCUSSION.

*Dr. Kime:* I have had no practical experience with electrolysis in this class of work, but for years I used it in gynecological work, but, as the author stated, it is a very tedious method, and I have since discarded it for other line of treatment. However, in his line it is different. Many things in my line could be treated by it, but other methods have superseded it. The paper is rather interesting, and the line brought out of the application to epithelioma is worth remembering when we cannot remove it by other methods. Some other troubles of malignant growths have of late been removed by this method.

### REPORT OF CASES.

*Dr. Duncan:* I have no case that I want to report, except that I had a call to-day to see a child, and the only object in calling me was to get me in a position to sign a death certificate. It is a peculiar case. It was the patient of another physician who was called out of the city. It was a little child six weeks old with a very extensive cleft palate involving the upper lip and one side of the nose, and the other side of the nose has no opening at all, but is entirely closed up. The child was dying from inanition, as it could not take any food. They had tried to feed it, but could not accomplish anything. They simply wanted a doctor to write a death certificate. As to the cause of death, I suppose I would have to write a certificate of death from inanition.

*Dr. Kime:* I have nothing of especial interest to report, but I recall to mind a case which was of interest to me. It was a case of labor to which I was called one Saturday night after dark. I found the cervix dilated to about the size of a half dollar and seemed to have a foot presentation. The pains were very slight. By external palpation I thought I could map out the fetus to some extent and the head was in the right hypochondriac region. I tried to change the position of the child, but could not do so on account of the pains. I was called early the next morning, as she was having hard labor pains, and I cleansed my hands and made examination and found the membranes had ruptured and a foot presenting. A few hard pains brought the buttocks down, and I had the patient moved around to the side of the bed and quickly disinfected my forceps and applied them and delivered the head. There was nothing of especial interest after this except the course of fever which followed. After labor she had continual fever for about a week and then the temperature dropped down to 99½ and normal in the morning. After that she had a severe headache every other day. This was followed by a chill, and the temperature went up to 103. I washed out the uterus and gave ten grains of quinine and the temperature was about normal the next morning. The drainage then became obstructed in some way and the temperature went up. After this was relieved she took cold and had pain in her left breast and temperature went to 102 again. After this it went down to normal and has been for last four or five days. She complained of the drainage-tube, and this morning I put in a smaller drainage-tube, and this afternoon the temperature was 102 and severe headache. It has been an interesting case to me all along.

Also another case had fever both before and after confinement, and two weeks after confinement gave instructions to use vaginal syringe, and she used a fountain syringe with a valve in the bag and there was some air in the syringe and this was carried up into the uterus. There was some ulceration of the cervix. After using the syringe her temperature went up to 103. I used drainage and washed out and the temperature went down to normal. She objected to drainage, and I substituted a very small tube, and it did not drain well, and she had two chills the second day afterward and temperature went up to 103. I put in the tube and gave quinine and the temperature came down and did not go up again.

In another case of another physician the patient went along all right for two weeks after confinement, and she was instructed to use a vaginal douche as she was having a discharge. She used a syringe that would pump up air. She used it at night and immediately following its use she had a chill and the temperature went up the next night to 103 and 104. I went down to see the patient and found the uterus larger than it should be, and on the right side the tube was one and a half inches in diameter. I used drainage and moved the bowels thoroughly, and she gradually improved for four or five days and then, for some cause, she had the same relapse. I continued the drainage, and then hot douches and glycerin applications, and she has been getting along nicely. These are two cases in which the douche has been used and caused trouble, and are the only two cases in my practice that I ever had any trouble from the vaginal douche, and I attribute it to those conditions mentioned.

*Dr. Duncan:* Did you think that the air used in the last case caused the fallopian tube to become an inch and a half in size in such a short time? Was that not the previous condition?

*Dr. Kime:* The patient formerly had some tubal trouble existing in that side before her pregnancy, but in this case there is no question that the air and water being carried up there and probably forced out into the pelvis had produced a pelvic cellulitis.

*Dr. McRae:* I have had recently two cases which were interesting on account of their rarity. In neither case did I make diagnosis prior to operation. One was the case of a gentleman from

Columbus, 67 years of age, having a large abdominal tumor which every surgeon who saw it diagnosed as I did, that is, as malignant disease, as the age of the patient, the general run-down condition, the appearance of the tumor and everything indicated malignancy. Dr. Cooper saw it and thought it was malignant. I thought it was probably carcinomatous. I suggested exploratory operation and I thought it might probably develop a condition which would be susceptible to treatment. There was no history of diarrhea but marked constipation which was difficult to relieve. Had been constipated for several months. The tumor was in the umbilical region and about the center of the abdomen. Upon opening the abdomen the cystic character of the tumor was apparent, but I still thought I would find it to be malignant. Upon examination I found it was retroperitoneal and completely filled in around the spleen, and I then thought it was probably a pancreatic cyst and a chemical examination showed it to be of this character. After the operation I introduced drain and the patient recovered, but still has a little sinus. The discharge has diminished and now there is very little. The discharge usually ceases entirely and the patient will probably get well. As to the rarity of this trouble, Dennis's surgery gives only fourteen cases of operation on record. I think Keen has probably operated once since that report, making fifteen cases, and my case is the sixteenth.

The other case was more puzzling. The man had recently come from Philadelphia, and it seemed to be a typical case of gall-stone in the gall-bladder. There was a cystic tumor and hepatic disturbance extending over three or four years, with a history of bilious fever for two months previous. I made a diagnosis of gall-stone with distended gall-bladder. I operated two days later and found that there was no solid body in the gall-bladder, but the right lobe of the liver was very hard. The patient was 34 years of age, and it did not seem to be malignant disease. The omentum over that portion of the liver was adherent. I passed a small canula into the liver and let out a little fluid which looked like serum and not like bile. I was puzzled over this condition and was undecided what was best to do. I stitched the parietal peritoneum to the liver, leaving the abdominal wall uncovered by peritoneum and then packed the space. On the third day I removed this packing and made an opening into the liver substance and removed thirty gall-stones from the liver itself. At the next dressing I got out three gall-stones, and at the third dressing removed ten and finally removed fifty-two. They were all beautiful specimens and were embedded in the liver substance. I have found out since that they are occasionally found in the liver substance, but I was not prepared for what I found in that case. It was of such interest to me that I thought it would probably interest the society. The patient is up and has good appetite and is gaining flesh and has no trouble since the operation, with the exception of taking a little cold and had a little rise of temperature for one day. He still has a sinus which is discharging a little mucous material and a very little bile. There has never been very much bile.

*Dr. Hutchins:* I would like to ask the doctor whether the stones were encysted?

*Dr. McRae:* The stones had formed quite a space in the liver. There was a cavity as large as an English walnut which was filled and packed in with them.

*Dr. Duncan:* Are the stones characteristic of biliary calculi?

*Dr. McRae:* They are very typical and I have crushed two or three and examined them carefully.

*Dr. Hutchins:* I would like to mention one of several cases upon which I have operated lately. This was a case of cancer involving the outer part of both the upper and lower lip and the corner of the mouth nearly down to the border of the jaw where the facial artery crosses. This part had sloughed out entirely, leaving an opening. It was thought best to first use the caustic potash, which was done under chloroform. As soon as the eschar had sloughed out clean the patient was given chloroform again and the diseased tissue dissected out, beginning at the facial artery, cutting this artery first to prevent further hemorrhage and the dissection was carried in to include part of the toothless gum and then carried up to near the center of both the upper and lower lip, the section removed en masse. The corners of the upper and lower lips, or remains of them, were stitched back on the raw surfaces to give the mouth the proper width, and the tissues over the chin were dissected loose as far as the mental artery on the opposite side, not cutting this artery. The wound was closed over the jaw bone, a portion of the fresh cut flap being left to make a new corner to the mouth. There was some odor after two days from the inside of the mouth. It was necessary to remove the dressing. With good care we succeeded in getting perfect first intention on the upper lip, and first intention but with a slight depression or furrow on the lower or chin part. The glands did not seem to be involved, although I may have trouble with them yet. The patient was very old and had a fatty heart, and came the nearest to death on the table that I have yet had.

